# Reduced glutathione and glutathione disulfide in the blood of glucose-6-phosphate dehydrogenase-deficient newborns

**DOI:** 10.1186/s12887-017-0920-y

**Published:** 2017-07-20

**Authors:** Zhen-hua Gong, Guo-li Tian, Qi-wei Huang, Yan-min Wang, Hong-ping Xu

**Affiliations:** 1 Department of general surgery, Shanghai Children’s Hospital, Shanghai Jiao Tong University, road, West Lane 1400, number 24. Shanghai, Beijing, 200040 China; 2 Neonatal screening center, Shanghai Children’s Hospital, Shanghai Jiao Tong University, Shanghai, China; 3 Department of neonatology, Shanghai Children’s Hospital, Shanghai Jiao Tong University, Shanghai, China

**Keywords:** Glucose-6-phosphate dehydrogenase deficiency, Blood, Glutathione, Tandem mass spectrometry

## Abstract

**Background:**

Glucose-6-phosphate dehydrogenase (G6PD) deficiency is commonly detected during mass screening for neonatal disease. We developed a method to measure reduced glutathione (GSH) and glutathione disulfide (GSSG) using tandem mass spectrometry (MS/MS) for detecting G6PD deficiency.

**Methods:**

The concentration of GSH and the GSH/GSSG ratio in newborn dry-blood-spot (DBS) screening and in blood plus sodium citrate for test confirmation were examined by MS/MS using labeled glycine as an internal standard.

**Results:**

G6PD-deficient newborns had a lower GSH content (242.9 ± 15.9 μmol/L)and GSH/GSSG ratio (14.9 ± 7.2) than neonatal controls (370.0 ± 53.2 μmol/L and 46.7 ± 19.6, respectively). Although the results showed a significance of *P* < 0.001 for DBS samples plus sodium citrate that were examined the first day after preparation, there were no significant differences in the mean GSH concentration and GSH/GSSG ratio between the G6PD deficiency-positive and negative groups when examined three days after sample preparation.

**Conclusion:**

The concentration of GSH and the ratio of GSH/GSSG in blood measured using MS/MS on the first day of sample preparation are consistent with G6PD activity and are helpful for diagnosing G6PD deficiency.

## Background

Glucose-6-phosphate dehydrogenase (G6PD) deficiency, an X-chromosome-linked genetic disorder, is the most prevalent mutation in humans, affecting more than 400 million people worldwide [1]. This disorder is characterized by decreased activity of the G6PD enzyme, which is the central factor of the antioxidant defense system in red blood cells (RBCs). The enzyme is responsible for maintaining high levels of reduced glutathione (GSH) and nicotine adenine dinucleotide phosphate (NADPH), which protect the cell from the oxidative damage caused by reactive oxygen species. Because G6PD-deficient RBCs are unable to generate NADPH through other pathways, these cells lack the ability to tolerate excessive amounts of oxidative stress [2, 3]. The most common clinical manifestation associated with G6PD deficiency is hemolytic anemia, which is generally triggered by the intake of oxidative drugs or foods. Occasionally, the defect can result in such complications as kidney failure, severe neonatal jaundice, or gallstones, and may require blood transfusion [4].

The diagnosis of G6PD deficiency is commonly based on the results of a fluorescent spot test for NADPH generation and a quantitative spectrophotometric assay of G6PD activity. Although these tests provide some perspective on the severity of a patient’s clinical symptoms, they are based on measurements of G6PD specific activity under normal conditions, and do not reflect the status of the patient’s antioxidant defense system, predominantly GSH/glutathione disulfide (GSSG). Furthermore, such tests also do not specifically predict the dynamic response of metabolite and enzyme activity levels in RBCs during the intake of oxidative agents. Indeed, not all G6PD-deficient patients detected via neonatal screening will develop neonatal jaundice or hemolytic anemia, even when oxidative drugs or foods are consumed [4, 5].

GSH is the major low molecular weight thiol in cells, with intracellular concentrations typically in the millimolar range. GSH acts as a recyclable antioxidant through the formation of glutathione disulfide (GSSG) and subsequent enzymatic reduction by glutathione reductase. Due to its relatively high concentration within cells and favorable reaction rates, GSH is a strong antioxidant in vivo [6]. All of the reduced GSH present in plasma (and other extracellular fluids) is of intracellular origin. However, the physiological pathways that regulate the supply of GSH to extracellular fluids are complex, as several organs may contribute to extracellular GSH to varying extents [7, 8]. RBCs can synthesize GSH from cysteine, glycine, and glutamic acid because these cells contain all of the enzymes necessary for GSH biosynthesis, and a significant percentage of RBC GSH is produced de novo daily, significantly contributing to the plasma pool of GSH [9]. Although plasma lacks reductases and coenzymes, such as NADPH, that provide reducing equivalents to reduce disulfides to thiols [10], both GSSG and glutathione conjugates (GS-X) are actively exported from RBCs when their intracellular concentration is high [11, 12]. This de novo re-synthesis may balance GSH loss due to GSSG and GS-X export and is regulated by a feedback mechanism [13].

Changes in redox state are commonly used as an index of oxidative stress within biological systems, and numerous methods have been established to quantify GSH and GSSG levels. The majority of techniques use reverse-phase chromatography for separation, with detection is based on the UV absorbance of the compound or the fluorescence of an adduct [14]; liquid chromatography coupled with mass spectrometric detection is also utilized to assay a variety of biological sources [15–17]. However, no tandem mass spectrometry (MS/MS) method that is suitable for screening or detecting G6PD deficiency is available for the quantification of these compounds in the blood. In the present study, we developed a high throughput MS/MS method for diagnosing G6PD deficiency in patients using dry-blood-spot (DBS) samples.

## Materials and methods

### Materials

GSH was obtained from AMRESCO (Solon, OH, USA). NeoBase non-derivatized MS/MS kits were purchased from PerkinElmer (Boston, MA, USA) and included isotopically labeled glycine (^15^N, 2-^13^C-glycinem, 762.2 μmol/l as the working solution) as an internal standard. The DBS extraction solution and the flow solvent were composed of methanol(1000 ml),water (2 ml), and formic acid(0.2 mmol/L).

The endogenous concentration of GSH in whole blood by this method is as low as 200 μmol/L, and as high as 500 μmol/L. The GSH recovery test samples were prepared with the extraction solution. GSH was added to vials containing 0.1 ml of extraction solution to produce GSH concentrations of 0,200,400,600,800,1000,2000,and 4000 μmol/L. All of the samples were dropped onto S&S Grade 903 filter paper using a 100-μl pipette without overlap, dried at room temperature, prepared in dry-spot, and stored at 4 °C until the MS/MS analysis.

### Methods

The blood samples for newborn G6PD screening, including 12 positive and 623 negative, were collected by puncturing the heel on the third to seventh day of age. The blood was dropped directly onto S&S Grade 903 filter paper without overlapped by another drop of blood and dried at room temperature. The DBS samples were transferred to our newborn screening center and stored for 2 to 4 days at 4 °C. A quantitative spectrophotometric assay of G6PD activity was used for G6PD deficiency screening (Neonatal G6PD KIT, PerkinElmer Life and Analytical Sciences; WallacOy, Turku, Finland).

Thirty seven blood samples were collected from the veins of neonates who tested positive during the G6PD screening (These 37 samples were not all from the 12 babies above, other patients were added). Thirty seven control blood samples from their parents and from 21 neonates control without G6PD deficiency were also intravenous collected. In each case, the blood was drawn into a tube containing sodium citrate as an anticoagulant and mixed immediately. The blood samples were transferred to the laboratory within 3 h and dropped onto S&S Grade 903 filter paper using a 100-μl pipette without overlap and dried at room temperature for 6–24 h until examination by MS/MS. The G6PD activity for the confirmation test was measured immediately when the blood arrived at the laboratory using a method that measures the G6PD/6-phosphogluconate dehydrogenase (6PGD) ratio [18]. The clinical and neonatal samples were obtained from maternity hospitals and the Children’s Hospital of Shanghai. This study approved by the ethics committee of the participating hospitals.

We extracted GSH and GSSG, use with one 3 mm DBS disk per analysis, using 100 μl extraction solution containing isotopically labeled glycine working solution (11:1) per sample in 96-well plate (NUNC),incubating in 45 °C, shaking at 700 rpm for 45 min. The extractions were transfer to another 96-well plate for mass spectrometry analyses.

**Fig. 1:**
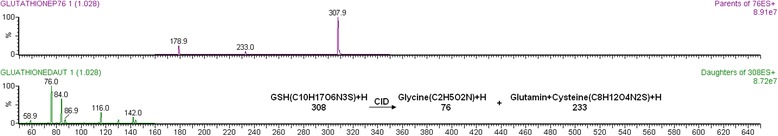
The MS/MS fragment ions assumed and fragment generated by collision-induced dissociation of GSH. The MS scans of the GSH solution show a peak at m/z 307.9(GSH307+H1). The product scan of m/z 308 shows a peak at m/z 76 (glycine75+H1^+^)

**Fig. 2:**
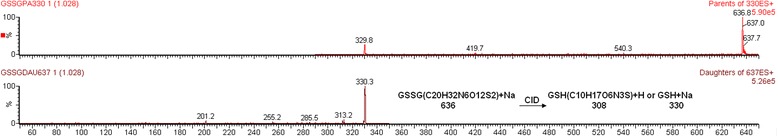
The MS/MS fragment ions assumed and fragment generated by collision-induced dissociation of the GSSG. The MS scans of the GSSG solution show a peak at m/z 636.8 (GSSG613 + Na23^+^). The product scan of m/z 636 shows a peak at m/z 330 (GS 307 + Na 23^+^)

Mass spectrometry analyses were performed using a Waters MICROMASS Quattro micro™API, (Manchester, UK). The electrospray needle was maintained at +3.5 kV, and the desolvation temperature was set to 350 °C. The desolvation gas flow was 650 L/Hr, the cone potential was 30 V, the collision energy was 3.0 eV, and the dwell time was 50 ms. Nitrogen, the sheath gas, was set at 50 units. The collision gas used was argon. The temperature of the heated capillary was maintained at 120 °C. GSH and GSSG were quantified in multi-reaction-monitor (MRM) mode using positive electrospray ionization mass spectrometry. The strongest MRM signals for GSH and GSSG were selected for quantification. Their fragment ions assumed and generated by collision-induced dissociation of the [GSH + H] ^+^ (Fig. 1)and [GSSG + Na]^+^ions (Fig. 2) were observed in product scans. The settings (precursor ion → fragment ion) for the target analytes were GSH m/z 308.0 → 76 and GSSG m/z 636.8 → 330. The setting for the isotopically labeled internal standard ^15^N, 2-^13^C-glycine was m/z 78 → 32 [19, 20]. The samples were delivered using an HPLC pump (Waters 1525 μBinary, MA, USA) equipped with a 20-μl sample loop. The samples were run at 116 μl/min from 0 to 0.23 min, at 20 μl/min from 0.24 to 1.35 min, and at 600 μl/min from 1.36 to 1.7 min. The concentration was calculated using Masslynx software, version 4.0 (Waters, Milford, MA, USA) by combining the intensities of the m/z 308.3 → 76 peak for GSH, the m/z 636.8 → 330 peak for GSSG, and the m/z 78 → 32 peak for the internal standard (^15^N, 2-^13^C-glycine) from 0.4 to 1.3 min, as measured as peak areas, for each sample. The serum concentrations were determined using the following formula: GSH = hematocrit × coefficient × Intensity m/z 308.3 → 76/78 → 32 + parameter; GSSG = hematocrit × coefficient × Intensity m/z 636.8 → 330/78 → 32.

All analyses were performed with SPSS 19.0 (SPSS Inc. Chicago, IL, USA). Differences of mean were tested by the one-way ANOVA among three groups and by independent *t* test between two groups. Significance was accepted at a *P* value of ≤0.05. Linear regression of the GSH/glycine-IS peak area ratio vs. the concentration of GSH added, and the ROC Curve of GSH/GSSG ratios to predict G6PD deficiency were done.

## Results

### MS/MS of GSH and GSSG

Based on the MS results for the product and precursor scans of the GSH (Fig. 1)and GSSG solutions (Fig. 2), we decided to use an MRM of m/z 308.0 → 76 for GSH, m/z 636.8 → 330 for GSSG, and m/z 78 → 32 for isotopically labeled glycine (^15^N, 2-^13^C-glycine) as an internal standard. We also combined the intensity of scans as the peak area for each analyte.

### Linearity

**Fig. 3:**
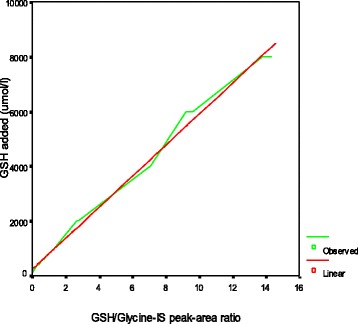
Linear regression of the GSH/glycine-IS peak area ratio vs. the concentration of GSH added

Calibration curves were obtained by linear regression based on a plot of the GSH/glycine internal standard peak-area ratio(x) vs. the concentration(y) of added GSH (Fig. 3). The concentration range used for the added GSH was 200–4000 μmol/L. The slope was 570 μmol/L, the intercept was 200 μmol/L, and the squared correlation coefficient was 0.994, *p* < 0.0001. We used the formula Y = 762 μmol/L × intensitym/z 308.3 → 76/78 → 32 + 200 μmol/L(hematocrit0.5 is assumed)to calculate the concentrations of GSH in the blood samples. The value used to represent GSH/GSSG was the peak area ratio of these compounds.

### Precision, accuracy, and recovery

The recoveries of GSH added DBSs were determined at nine concentrations and were found to be in the range 90 to 126% for GSH in the concentration range 200–4000 μmol/L (Table 1) when examined on the first day (within 24 h) after sample preparation. However, the GSH recovery gradually decreased when the samples were examined on the second day and thereafter (Table 1).

**Table 1:** Recoveries and precision of GSH measured on the 1st and 3rd days after sample preparation

	Day 1 (*n* = 6)			Day 3 (*n* = 6)	
GSH Added (μmol/L)	GSH Measured (μmol/L)	CV %	GSH Recovery (%)	GSH Measured (μmol/L)	GSH Recovery (%)
0	213.2 ± 7.0	3.2		207.7 ± 3.62	
100	211.6 ± 1.4	0.6		208.4 ± 1.12	
200	253.4 ± 9.2	3.6	126.7 ± 4.6	214.7 ± 5.1	107.3 ± 2.6
400	397.9 ± 27.4	6.8	99.5 ± 6.8	257.3 ± 5.7	64.3 ± 1.4
600	584.3 ± 41.3	7.0	97.5 ± 6.9	345.4 ± 17.2	57.5 ± 2.8
800	726.5 ± 13.1	1.8	90.8 ± 1.6	488.6 ± 14.4	61.0 ± 1.8
1000	939.4 ± 6.0	0.6	93.9 ± 0.6	678.9 ± 16.5	67.9 ± 1.6
2000	1748.8 ± 27.4	1.5	87.4 ± 1.4	1574.8 ± 121.9	78.74 ± 6.10
4000	4234.6 ± 6.4	0.2	105.9 ± 0.2	3745.1 ± 488.4	93.6 ± 12.2

### GSH and GSH/GSSG in confirmed G6PD-deficient newborns

The confirmed G6PD-deficient newborns had lower blood levels of GSH and lower GSH/GSSG ratios than the neonatal and parental controls (Table 2). All the blood samples for confirming test contained sodium citrate were prepared in DBS and examined on the first day. The parents should carry the allele conferring G6PD deficiency, and some exhibited decreased, albeit only slightly, G6PD activity, led to the GSH concentrations and GSH/GSSG ratios in the parent group were higher than patient group and lower than the normal control group.

**Table 2:** The GSH content and GSH/GSSG ratios in confirmed G6PD-deficient newborns, neonatal controls, and parental controls

	Number	GSH (μmol/L)	GSH/GSSG
G6PD-deficient newborn	37	242.9 ± 15.9	14.9 ± 7.2
Neonatal control	21	370.0 ± 53.2	46.7 ± 19.6
Parental control	37	287.5 ± 48.2	27.3 ± 16.1
*P*		0.001	0.001

On the ROC Curve for GSH/GSSG ratios predicting G6PD deficiency, the area under the curve was 0.679, *p* = 0.003. GSH/GSSG ratios lower than 30 was used as cut-off value to determine state of oxidative stress, which caused by G6PD-deficiency in newborns, the sensitivity is 91.9%; and 1-speficity is 79.3%.

### GSH levels and GSH/GSSG ratios in newborns screening positively for G6PD deficiency

The GSH levels and GSH/GSSG ratios in DBS samples are routinely examined in neonatal disease screening by combining the detection of amino acids and acylcarnitines using MS/MS. These DBS samples were prepared in maternity hospitals without the use of sodium citrate and examined at least three days after collection. The activity of G6PD in the DBS samples was examined on the second day after the GSH levels were examined using MS/MS. The activity of G6PD was positively correlated to the GSH/GSSG ratio (*R* = 0.213, *p* < 0.001 *n* = 278), though no significant positive relationship with the GSH content was observed (*R* = 0.027, *p* > 0.05, *n* = 278). However, there were no significant differences in the mean GSH concentrations and the GSH/GSSG ratios between the G6PD deficiency-positive group and the G6PD deficiency-negative group (Table 3).

**Table 3:** G6PD activity, GSH levels, and GSH/GSSG ratios in test samples from newborns that screened positive or negative for G6PD deficiency

Screening for G6PD deficiency	Number	G6PD activity	GSH (μmol/L)	GSH/GSSG ratio
Positive	12	1.12 ± 0.55	376.6 ± 56.3	44.5 ± 19.5
Negative	623	4.59 ± 1.39	413.6 ± 84.5	52.8 ± 20.9
*P*		0.001	0.13	0.17

## Discussion

Human blood contains GSH, and in addition to diet and aging, many pathological conditions influence the GSH concentration [21, 22]. The whole blood and plasma concentrations of GSH differ, and the concentrations measured differ within the same group of people depend on the method used [23–26]. We found whole blood GSH concentrations to be 100–500 μmol/L, which is 100 times higher than in plasma and approximately half the GSH concentration found in red blood cells (data not shown). Although consistent with another study [26], the values obtained in this study were slightly lower than those found by some other studies [22, 25]. This discrepancy might have occurred because our samples were collected from neonates or adults with alleles conferring G6PD deficiency; further, we only selected the MRM m/z 308.3 → 76 peak to detect GSH and combined the MRM signals rather than the GSH peak area. This simplified method made it possible to combine the neonatal disease screening system for detecting amino acids and acylcarnitines with MS/MS.

GSH is an unstable molecule. We used labeled glycine (^15^N, 2-^13^C-glycine m/z 78 → 32) rather than labeled GSH as an internal standard. The Glycine + H^+^fragmentis an ion that is generated by the collision-induced dissociation of GSH, and stable labeled glycine can more accurately reflect unstable GSH levels. The observed recovery of GSH levels decreased gradually after the GSH samples were prepared, which has also been reported by other studies that used reagents to ensure that GSH was not oxidized to GSSG or other GS-X compounds [27]. In our study, the GSH levels in blood that was mixed with sodium citrate (an anticoagulant) were more stable than those in blood collected by newborn heel puncture without the use of anticoagulant. This finding may be one of the reasons why the GSH measured in the neonatal screening DBS sample did not reflect the activity of the G6PD enzyme; indeed, the DBS samples were usually measured at least three days after they were collected.

A decrease in the levels of GSH, the reduced form of glutathione, which was accompanied by an increase in the basal level of GSSG, has been correlated to a lower susceptibility of RBCs to osmotic hemolysis [5, 28]. The ratio of GSH/GSSG measured in blood is different from that measured in other tissues and also differs depending on the technique [17, 23, 27]. In our study, the ratio of GSH/GSSG in whole blood was 10–100 times higher than that observed in other reports. One reason for this discrepancy is that we did not use an antioxidant to treat the sample. The GSH/GSSG ratio in the whole blood (with added sodium citrate) of the G6PD deficiency group was lower than that of their parents, and the latter was lower than that of the normal neonatal group. These findings indicate that the ratio of GSH/GSSG can be correlated to the activity of G6PD in blood that contains sodium citrate and is examined on the first day of sample preparation. Therefore, we can use MS/MS to measure GSH/GSSG ratios to confirm G6PD deficiency or predict that patients are more likely to suffer from hemolytic anemia because they already have a lower antioxidant status.

## Conclusion

The concentration of GSH and the ratio of GSH/GSSG in the blood, as measured using MS/MS on the first day of sample preparation, are consistent with the G6PD activity and are helpful for diagnosing G6PD deficiency.

## Abbreviations

DBS: dry-blood-spot
G6PD: Glucose-6-phosphate dehydrogenase
GSH: reduced glutathione
GSSG: glutathione disulfide
MS/MS: tandem mass spectrometry
